# Toward a Better Understanding of Cardiovascular Risk in the Transgender and Gender-Diverse Community: A Global Call to Action

**DOI:** 10.5334/gh.1300

**Published:** 2024-02-29

**Authors:** Jorge Eduardo Cossío Aranda, Viveka Jain, Lourdes M. Figueiras-Graillet, Alexandra Arias-Mendoza, Julio López Cuéllar, Ana Berni Betancourt, Álvaro Sosa Liprandi, Fausto Pinto, Jean-Luc Eiselé, Daniel José Pineiro

**Affiliations:** 1 Mexican Society of Cardiology, Juan Badiano 1, Col. Section XVI, Tlalpan, 14080 Mexico City, Mexico; 2 World Heart Federation, Rue de Malatrex 32, 1201 Geneva, Switzerland; 3 Inter-American Society of Cardiology, Juan Badiano 1, Col. Seccion XVI, 14080 Mexico City, Mexico

**Keywords:** transgender health, cardiovascular disease, minority stress theory

## Abstract

On World Heart Day 2022, the Mexican Society of Cardiology, the Inter-American Society of Cardiology, and the World Heart Federation collaborated on a public call to action regarding the increased risk of adverse cardiovascular health outcomes in transgender and gender diverse (TGD) individuals. The aim of this article is to unpack the numerous factors that contribute to this, such as the social stigma faced by members of the TGD community, their reduced access to clinical care, and the scarcity of research regarding the unique needs of their community, which makes it difficult for clinicians to provide individualized medical care. Decreasing the incidence of adverse cardiovascular events among TGD individuals requires interventions such as educational reform in the medical community, an increase in inclusive research studies, and broader social initiatives intended to reduce the stigma faced by TGD individuals.

## Background

Cardiovascular diseases (CVDs) are the leading cause of death globally, representing nearly 32% of deaths annually [1]. Systemic health disparities that cause the burden of disease to be disproportionately heavier on the shoulders of minority groups have been well documented in scientific literature. Data collected from American adults through the Behavioral Risk Factor Surveillance System (BRFSS) and the All of Us research program demonstrate that gender significantly modulates the odds of developing cardiovascular health problems, and that gender non-conforming individuals are at a disproportionately high risk for developing disorders [2,3,4]. For example, BRFSS data shows that the adjusted odds ratio (AOR) for coronary heart disease/myocardial infarction comparing transgender women to cisgender women was 2.07 (95% CI 1.37, 3.13) [4]. There is also evidence of higher rates of risk behaviors in the transgender community. Transgender females have greater odds of engaging in heavy drinking than their cisgender counterparts (AOR 1.81, 95% CI 1.26, 2.60), transgender men are more likely to engage in no exercise than cisgender men (AOR 1.85, 95% CI 1.31, 2.62) and have higher odds of suffering from multiple chronic diseases, including type 2 diabetes and arthritis (AOR 1.88, 95% CI 1.32, 2.67) [4]. Though the incidence of CVD in the TGD community has the potential to be mitigated with preventative medicine and lifestyle modifications that are generalizable to the broader population, they also face a unique set of sociocultural factors that warrant clinical consideration [2,5]. The reasons for the increased incidence of CVD among the TGD community are broad-ranging, and the solution to this problem requires both interdisciplinary inquiry and multisectoral collaboration.

This is the subject of a public call to action that was put forth by the Mexican Society of Cardiology, the Inter-American Society of Cardiology, and the World Heart Federation (WHF) on September 29, 2022 (the date of World Heart Day) [5]. In keeping with WHF’s mission to provide cardiovascular health for all, this declaration synthesized information regarding the increased incidence of adverse cardiovascular health events in the TGD community in Latin America and served as a call-to-action for cardiovascular health-focused organizations to work to close this crucial health disparity [5]. The objective of this report is to supplement this call to action by providing a global perspective on health disparities in TGD communities with a focus on disparities in cardiovascular health, exploring the multiple facets of this issue, and articulating some priorities for the scientific community in the process of advancing towards the goal of providing equitable healthcare for all.

## The Sociodemographic Context of TGD Cardiovascular Health

The social determinants of health are the social, political, economic, and environmental factors that influence an individual’s ability to maintain good health. Among the key social determinants of health are socioeconomic status, race/ethnicity, gender, and housing situation [6]. While interpersonal rejection and financial insecurity due to these factors can predispose an individual to CVD by directly impeding access to healthcare services, the physiological stress and inflammation caused by social determinants of health can also further negative health impacts [7]. For instance, residence in disadvantaged neighborhoods that have high poverty rates, food insecurity, and low levels of social cohesion has been found to be related to increased levels of inflammatory biomarkers such as IL-6, TNF-α, and IL-1β, the elevation of which has been linked with the development of CVD [7]. Thus, it is important to consider the unique intersections of sociodemographic challenges faced by the transgender community in order to understand their elevated CVD risk.

Survey data from a community-based participatory research study conducted in Puerto Rico revealed that 65.4% of transgender respondents had experienced public harassment due to their gender identity at least once and 55.8% had experienced intimate partner abuse [8]. A cross-sectional study conducted among California middle and high schoolers demonstrated that transgender youth are 2.5 to 4 times more likely to develop a substance use disorder than their cisgender counterparts, and BRFSS data revealed that transgender adults are at a higher odd of suffering from depressive disorder (for transgender women, AOR 2.02, 95% CI 1.52, 2.69; for transgender men, AOR 3.14, 95% CI 2.07, 4.77) [4,9]. These findings reflect the broader consensus that, relative to other demographic groups, members of the LGBTQIA+ community are predisposed to experiencing public harassment or violence due to their gender expression and are at increased risk of developing mental health complications and substance abuse disorders, all of which contribute to increased CVD risk [8,9].

Another LGBTQIA+ issue that is especially significant for the TGD community is identity development. The process of creating an identity as a transgender person that is both internally and externally acceptable is extremely nuanced and is heavily dependent on the perception of others. Transgender individuals often face external pressure to conceal their identity and “pass” as cisgender, which can help them avoid discrimination but may alienate them from in-group solidarity found within the LGBTQIA+ community [10]. These barriers to forming effective social relationships can further increase stress faced by TGD individuals, predisposing them to developing CVD.

In the Transition Experience Study, the lived experience of the social and medical gender transition was examined through surveys and interviews with a sample of transmasculine individuals living in the United States of America [11]. Residence in regions where the geopolitical climate is perceived to be progressive was associated with less stress in TGD individuals, as evidenced by linear regression models of the relationship between the progressiveness of a region and biomarkers of allostatic load (p = .001). Another protective factor that was discussed in this study is sociodemographic advantage, which is the presence of factors that are socially acceptable (i.e., non-minority race, high socioeconomic status). In line with researchers’ hypotheses, sociodemographic advantage was also negatively correlated with markers of stress [11].

The concept that is often used to articulate the relationship between stress and adverse health outcomes in gender and ethnic minorities is the minority stress theory (MST). The Gender Minority Stress and Resilience Model visualizes MST by depicting distal stressors (i.e., gender-based discrimination) and proximal stressors (i.e., internalized transphobia), which converge and contribute to increased levels of stress in minority groups [2]. To expand on the MST and include consideration of various intersecting minority identities, the American Heart Association has developed the Intersectional Transgender Multilevel Minority Stress model, which relates health status to the “degree of stigmatization” (i.e., someone with multiple intersecting minority social identities will face a higher degree of stigmatization than someone with only one) [2].

This model, along with other available literature on this subject, illustrates the relationship between social stigma and cardiovascular health. Transgender individuals may face stigma at the individual level (i.e., internalized homophobia), the microsystem level (i.e., through enacted and perceived stigma in interpersonal interactions), and the macrosystem level (i.e., structural stigma) [2]. The negative health impacts of stigmatization can be counteracted with resilience promoting factors at each level (for example, improved community connectedness at the microsystem level) [2]. Interventions intended to address stigma beyond the clinic will likely be required to reduce the minority stress faced by the TGD community as well as the broader LGBTQIA+ community, which in turn will impact their cardiovascular health outcomes.

## Access to Quality Healthcare in the TGD Community

According to the most recent ACC/AHA guidelines for the primary prevention of cardiovascular disease, three central recommendations for patient-centered prevention of CVD are increased use of team-based care (which is the collaboration of multidisciplinary healthcare professionals on each case), shared decision-making between the provider and patient, and adequate consideration of the social determinants of health in developing treatment regimens [12]. All three of these recommendations rely on a healthcare workforce that is well-informed, free of prejudice, and easily accessible to all patients.

However, this is often not the case for TGD patients. A study from the U.S. demonstrated significant disparities in access to healthcare services between sociodemographic identities. White and cisgender patients have higher rates of health insurance and are more likely to have the financial capacity to pay for medical services (P = 0.01), and transgender patients are more likely to delay seeking medical attention (P < 0.001) and report negative experiences with medical providers (P < 0.001) [13]. These disparities result from a combination of sociodemographic factors such as income level and stigma, as well as a unique set of challenges faced by gender non-conforming individuals in the patient-provider relationship.

Additionally, there are currently no standardized methods to collect information about gender identity in patient histories, thereby reducing visibility of transgender patients in the healthcare system [14]. Along the same lines, healthcare professionals typically do not receive training on the specific needs of the TGD community, as existing medical education initiatives often focus on the LGBTQ+ community in a general sense [15]. The attitudes of many healthcare providers are another barrier to effective healthcare for the TGD community. When nursing, health sciences, and medical students at a public university in Istanbul were surveyed using the Hudson and Ricketts Homophobia Scale, most study participants were found to exhibit medium levels of homophobia [16]. Some transgender patients even resort to medical travel in pursuit of an accepting environment in which to undergo gender-affirming procedures [17]. For example, a 2010 study found that it is common for transgender women to undergo gender-affirming procedures in Thailand instead of in Australia, America, and Europe due to the inclusive and respectful environment that can be found in the Thai medical community [18].

These findings expose the general heteronormativity of the healthcare system. Homophobia is not only evident in individuals in the medical field, but it is also embedded in the system. Healthcare professionals are not taught to consider the gender identities of their patients when providing care, which often forces TGD patients to advocate for themselves to receive proper medical care.

Some publications provide guidance on how medical professionals can adopt a more informed and sensitive approach to patient care in the TGD community. A narrative review by Rosendale et al. (2018) provides a brief guide to gender-inclusive medical care for clinicians and includes recommendations such as using gender-neutral pronouns until a patient specifies their gender identity and taking an inclusive “anatomic inventory” including questions about whether patients have had gender-affirming surgery [14]. The Standards of Care for the Health of Transgender and Gender Diverse People (the most recent version, the SOC-8, was published in 2022) is a periodic publication outputted by the World Professional Association for Transgender Health (WPATH) that provides guidance for clinicians on how to care for the TGD community [19]. Widespread integration of research such as this into the education of healthcare professionals is crucial to making the clinical setting a safe space for TGD individuals.

## The Impact of Gender Affirming Hormone Therapy on Cardiovascular Health

Since the gender of a transgender individual differs from the sex that was assigned to them at birth, the incongruency between the gender they identify with and their physical characteristics can cause a type of psychological distress known as gender dysphoria. Gender-affirming hormone therapies (GHT) can mitigate these feelings by modifying a person’s physical characteristics to match their identified gender [20].

Research regarding the relationship between GHT and cardiovascular health suggests that estrogen therapy as administered to transgender women (women who were assigned male at birth) increases their risk for venous thromboembolism over 5-fold [21]. GHT for both transgender women and transgender men (men who were assigned female at birth) has been demonstrated to have a significant impact on blood pressure (contributing to an increase of 17.8 mmHg in transgender women and 13.4 mmHg in transgender men after two years of GHT) [21]. However, several findings regarding the influence of GHT on cardiovascular health and risk factors are inconsistent. For example, elevated body mass index (BMI) is a risk factor for the development of CVD, and the relationship between GHT and BMI has been explored in a handful of studies [2]. One systematic review revealed significant increases in the BMI of transgender men after they began GHT (1.3%–11.4%); however, a longitudinal study revealed no significant increases in BMI [2,22,23]. Hormone therapy also has also been shown to lead to increased triglycerides (~21.4 mg/dL, 95% CI, 0.14–42.6), higher LDL cholesterol (17.8 mg/dL; 95% CI, 3.5–32.1), and lower HDL cholesterol (–8.5 mg/dL; 95% CI, –13 to –3.9) [21]. However, according to 2014–2017 data from the BRFSS, there were no differences in self-reported hypercholesteremia between TGD and cisgender adults [2]. Further research is required to generate reproducible findings in the relationship between GHT and CV health.

One study investigated a potential mechanism for the relationship between hormone therapy and inflammation, as measured by systematic and endothelial biomarkers, platelet activation markers, and coagulation markers. The principal finding was that hormone therapy has been demonstrated to reduce inflammatory biomarkers (hs-CRP –66%, (95% CI –76; –53), VCAM-1–12%) and increase platelet activation markers (PF-4 +17%, (95% CI 4; 32), β-thromboglobulin +13%, (95% CI 2; 24)) [25]. Inflammation has a potent impact on cardiovascular health, and these findings describe a potential explanation for the biological basis of the relationship between GHT and CVD [25]. Additional research on the mechanism by which GHT impacts cardiovascular health is limited.

Studies on GHT are often limited in scope and generalizability. For example, they generally only include participants younger than 50 years of age, which means that the medical community is not well-informed on the influence of GHT throughout the process of aging. Additionally, many findings on the health impacts on GHT have the potential to be confounded by factors such as the rates of mental health disorders and substance abuse in TGD populations, which can also have a significant impact on CV health [24]. In a review analyzing the association between the route of administration of estrogen therapy and cardiovascular risk in transgender women, Miranda et al. (2022) identified a variability in estrogen formulation, dose, and treatment duration in the studies that were included [26]. The lack of stratification based on these factors during data collection introduces potential confounding of the results [26]. Issues that were mentioned in other papers include the need for large cohort studies and longer follow-up periods to determine the long-term impacts of GHT, and the lack of consensus on the appropriate control populations to be used in studies on TGD patients [24,27].

Inclusive research endeavors are crucial for the attainment of health equity, and the lack of research regarding gender affirming hormone therapies is likely to have an impact on the quality of medical care for the TGD community. The transgender community is under-represented in cardiovascular research, which does not allow for understanding health disparities related to transgender identity. To create a more gender-inclusive medical field, more data elucidating the unique risks faced by transgender patients is required.

## Conclusion

Higher rates of adverse cardiovascular health outcomes in the TGD community can be attributed to a range of factors, including social determinants of health, structural issues with the healthcare system, side effects from gender-affirming treatments, and a lack of research on the unique needs of this community. Dismantling the web of structural violence that has led to this elevated CVD risk in the TGD community requires interdisciplinary collaboration. For example, the homophobia among pre-health students described by Harmanci Seren et al. [16] is a microcosm of broader patterns of homophobia in their societies. Educational reform in health-oriented graduate schools is important to create a generation of medical professionals who are aware of the unique struggles of the TGD community, but social initiatives that aim to address homophobia and gender bias on a broader scale are just as significant to create a shift in the social commentary about this community. Healthcare professionals, cardiologists, and primary practitioners have the obligation to educate themselves about the transgender community and work to make their practice an inclusive and accepting environment. Additionally, to echo the calls to action in many of the studies discussed in this report, inclusive and holistic data on this subject are needed. Specifically, there is a lack of controlled clinical trials regarding the therapeutic applications and side effects of gender-affirming hormone therapy, as well as a paucity of studies intended to learn about the needs of the aging transgender community.

Ultimately, an overarching goal of all this work is to build trust among the TGD community in a healthcare system that has historically been heteronormative. Meaningful educational initiatives for healthcare professionals and the broader society is imperative to building trust in the transgender community, as are research efforts that spotlight marginalized populations and make their voices heard. Creating a society with lower rates of CVD and narrower health disparities among social groups is a huge undertaking, but one that can be made possible with meaningful and intersectoral collaboration.
